# Surgical Treatment of Atrial Fibrillation in Patients with Rheumatic Valve Disease

**DOI:** 10.21470/1678-9741-2017-0016

**Published:** 2017

**Authors:** Ernesto Koehler Chavez, Alexandre Siciliano Colafranceschi, Andrey José de Oliveira Monteiro, Leonardo Secchin Canale, Evandro Tinoco Mesquita, Clara Weksler, Odilon Nogueira Barbosa, Anderson Oliveira

**Affiliations:** 1 Instituto Nacional de Cardiologia (INC), Rio de Janeiro, RJ, Brazil.; 2 Universidade Federal Fluminense (UFF), Niterói, RJ, Brazil.

**Keywords:** Atrial Fibrillation, Ablation, Catheter Ablation, Rheumatic Disease

## Abstract

**Objective:**

To assess heart rhythm and predictive factors associated with sinus rhythm after one year in patients with rheumatic valve disease undergoing concomitant surgical treatment of atrial fibrillation. Operative mortality, survival and occurrence of stroke after one year were also evaluated.

**Methods:**

Retrospective longitudinal observational study of 103 patients undergoing rheumatic mitral valve surgery and ablation of atrial fibrillation using uni- or bipolar radiofrequency between January 2013 and December 2014. Age, gender, functional class (NYHA), type of atrial fibrillation, EuroSCORE, duration of atrial fibrillation, stroke, left atrial size, left ventricular ejection fraction, cardiopulmonary bypass time, myocardial ischemia time and type of radiofrequency were investigated.

**Results:**

After one year, 66.3% of patients were in sinus rhythm. Sinus rhythm at hospital discharge, lower left atrial size in the preoperative period and bipolar radiofrequency were associated with a greater chance of sinus rhythm after one year. Operative mortality was 7.7%. Survival rate after one year was 92.3% and occurrence of stroke was 1%.

**Conclusion:**

Atrial fibrillation ablation surgery with surgical approach of rheumatic mitral valve resulted in 63.1% patients in sinus rhythm after one year. Discharge from hospital in sinus rhythm was a predictor of maintenance of this rhythm. Increased left atrium and use of unipolar radiofrequency were associated with lower chance of sinus rhythm. Operative mortality rate of 7.7% and survival and stroke-free survival contribute to excellent care results for this approach.

**Table:** 

Abbreviations, acronyms & symbols	
AF	= Atrial fibrillation
AHA	= American Heart Association
COPD	= Chronic obstructive pulmonary disease
CPB	= Cardiopulmonary bypass
LA	= Left atrium
LV	= Left ventricular
NYHA	= New York Heart Association Functional
SAH	= Systemic arterial hypertension

## INTRODUCTION

Atrial fibrillation (AF) is the most common sustained arrhythmia in the United States, where a prevalence of 2.2 million people with AF is estimated^[1,2]^. The prevalence of AF increases with age. It is estimated that the incidence of AF doubles for each decade of adult life. AF affects 2.3% of individuals over 40 years and 5.9% of those over 65 years^[1]^.

Approximately 70% of individuals diagnosed with AF are between 65 and 80 years old^[1]^. In addition, it can also be considered an important risk factor for stroke^[3]^.

AF is an independent risk factor for mortality, with relative risk for all ages of 1.5 for men and 1.9 for women^[2]^.

In the last 20 years, hospital admissions related to AF increased by 66%^[4]^. In Brazil, AF is the 5^th^ largest cause of hospitalization in the Unified Health System (SUS - acronym in Portuguese) and affects 10% of the population served by the specialized department of cardiology in hospitals with a level of complexity of quaternary care^[5]^.

The high prevalence of this arrhythmia, the expenses with the health system and the high morbidity and mortality associated with that disease have justified the search for a better understanding of its pathophysiological bases and of new therapeutic approaches^[4]^.

Despite being considered a benign disease, AF is associated with significant morbidity and mortality due to three levels of sequelae:

- Discomfort and anxiety generated by irregular heartbeats;
- Hemodynamic compromise due to loss of atrioventricular synchrony;
- Blood stasis inside the left atrium, with greater vulnerability to systemic thromboembolism.

AF may be related to thromboembolism and stroke in 5-10% of high-risk patients^[6]^. The risk increases with age and the presence of concomitant structural heart disease. According to the American Heart Association (AHA), it is estimated that more than 70,000 cases of stroke are related to AF each year.

**Fig. 1:**
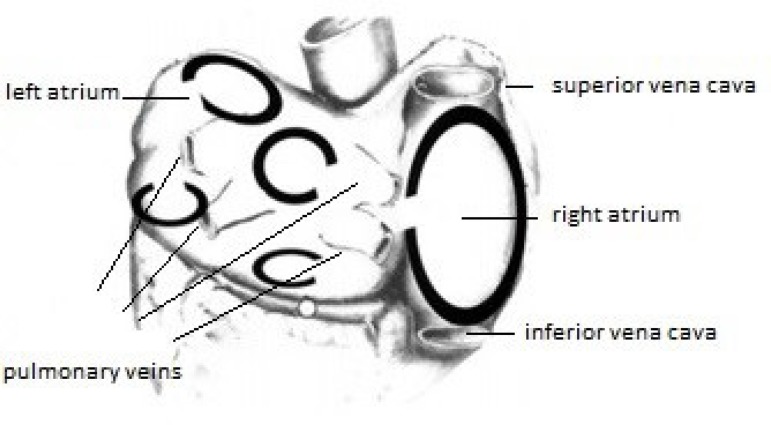
Atrial fibrillation (Reentry circuits). Adapted from Cox et al.^[12]^.

Up to the present time, the Maze surgery is the gold standard in the surgical treatment of AF, with success in healing AF greater than 90%^[7-11]^. The mechanism by which the procedure is able to prevent recurrence of AF is not yet conclusively established. However, the theory is that, with surgery, conduction blocks are created in the right and left atria, preventing macro-reentry circuits, responsible for the persistence of AF (Figure 1)^[12]^.

Maze surgery involves the creation of surgical incisions at strategic sites of both atria which are then re-sutured by creating electrical conduction block lines that prevent the formation and conduction of errant electrical impulses, driving the electrical impulse generated at the sinus node to the atrioventricular node. Maze surgery was designed to accomplish three objectives:

- Stop all reentry circuits that can lead to the development and maintenance of AF;
- Restore control of the heart rate to the atrial pacing complex;
- Allow the entire atrial myocardium to be activated to preserve the atrial transport function.

Maze surgery, when performed, is often concomitant with other cardiac procedures such as coronary artery bypass surgery, valve repair and/or replacement. Despite the effectiveness of Maze surgery, its completion is still restricted due to its morbidity related to the time required to perform the set of lesions, among others factors. Surgical risk increases due to the high times required of cardiac anoxia and CPB (cardiopulmonary bypass). Due to this, great effort is required to reduce the morbidity associated with this procedure^[10,11,13-15]^.

Recently, different sources of energy have been identified in order to reduce the time and morbidity of surgical procedures for the treatment of AF^[4,8,16]^. In the last 10 years, great advances have occurred in the development of different energy sources capable of creating electric conduction blocks in myocardial tissue. The application of endo- or epicardial radiofrequency is capable of producing myocardial necrosis that electrically isolates myocardial tissue in a manner similar to that demonstrated by classic Maze surgery (cutting and suturing)^[15,17,18]^.

The ideal set of lesions in the atrial myocardial tissue is not yet defined. Isolation of the pulmonary veins and excision of the left atrium are mandatory and seem to be associated with AF-free survival. Several methods including these components have corrected AF in 70% to 90% of patients. The importance of connecting lesions in the left atrium (e.g. between right and left pulmonary veins or left pulmonary veins to the mitral annulus) is uncertain. Atrial fibrillation arising from the right atrium is uncommon. Right atrial lesions are required only in patients with typical atrial flutter. As a result, isolation of the pulmonary veins combined with excision of the left atrium may represent the appropriate operation for most AF patients. However, additional studies are needed to test this hypothesis.

Records and retrospective analyzes have not shown an increase in surgical mortality when intervention on the mitral valve is performed concomitantly with atrial fibrillation ablation^[19]^. However, an increase in postoperative morbidity is observed^[2]^.

In Brazil, a large part of the population of patients in the Unified Health System requiring surgical intervention on the mitral valve has a diagnosis of rheumatic heart disease. In this clinical scenario, the impact of the use of bipolar radiofrequency devices in the surgical treatment of atrial fibrillation in operative mortality and in event-free survival is still to be defined^[20-22]^.

The main objective included the evaluation of the cardiac rate at hospital discharge and a one-year postoperative follow-up period as well as factors associated with the occurrence of sinus rhythm at the end of the first year with patients presenting rheumatic heart disease and indication for mitral valve surgery concomitant with surgical ablation of atrial fibrillation.

The secondary aim of this study is to describe operative mortality and one-year survival, as well as the incidence of stroke in the same period.

## METHODS

Observational, longitudinal and retrospective study. One hundred and three consecutive patients undergoing mitral valve surgery of rheumatic etiology with concomitant atrial fibrillation ablation using unipolar or bipolar radiofrequency were evaluated. Patients undergoing associated tricuspid repair were included. All patients were operated at the Instituto Nacional de Cardiologia (INC) between January 2013 and December 2014.

Patients undergoing other associated procedures such as aortic valve replacement or coronary artery bypass grafting were excluded. Reoperation procedures were also excluded.

Study performed in a Brazilian public institution in which patients are cared for by several surgical teams. Seven different surgeons participated in the surgical interventions as the first operator.

In all patients, electrical isolation of the pulmonary veins was performed in pairs (left and right). Atrial connection ablation lines (upper and/or lower) and the type of device used for ablation (uni- or bipolar radiofrequency) were chosen according to the preference of the main surgeon and the availability of the device at the institution. The closure of the left atrial appendage was also performed at the discretion of the surgeon, always through the endocardial route with continuous suture with 3-0 or 4-0 Prolene.

The pre-, intra- and postoperative clinical and surgical variables investigated were:

1. Age
2. Gender
3. Comorbidities: systemic arterial hypertension (SAH), diabetes, chronic obstructive pulmonary disease (COPD) and smoking.
4. New York Heart Association Functional Class (NYHA) functional class
5. Type of AF: paroxysmal, persistent or permanent (American Heart Association - AHA)
6. EuroSCORE
7. Time of fibrillation
8. Previous stroke
9. Left atrium (LA) size by echocardiography
10. Left ventricular (LV) ejection fraction by echocardiography
11. Cardiopulmonary bypass time
12. Time of myocardial ischemia

**Fig. 2:**
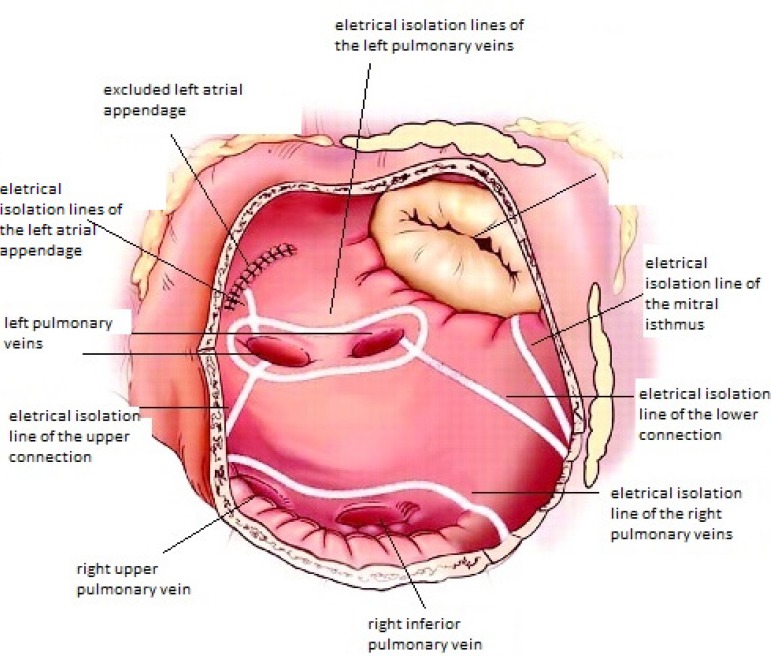
Lines of tissue lesion by radiofrequency in the left atrium. Adapted from Weimar et al. ^[23]^.

Ablation lines performed: isolation of the pulmonary veins in pairs; lesions in the posterior wall of LA; connecting line to the mitral valve annulus; cavo-tricuspid line (between the inferior vena cava ostium and the tricuspid valve annulus); cava-cava line (between the ostia of both venae cavae) (Figure 2).

Patients were followed-up postoperatively with visits scheduled for 1 month, 6 months and 12 months after surgery. During these visits, a non-blind investigator for the offered treatment has evaluated heart rate, medication use, thromboembolic events, need for percutaneous or surgical re-interventions.

The rhythm investigation was always performed with conventional CPB at each medical appointment and with 24h Holter.

The criterion of therapeutic failure was that of any irregular tachyarrhythmia lasting more than 30 seconds.

Table 1 shows the general characteristics of the patients analyzed.

**Table 1:** Characteristics of the patients studied.

Age (years)	
Minimum	23
Maximum	72
Mean ± SD	50.76±10.7
Gender	
Female	78 (76%)
Male	25 (24%)
**Comorbidities**	
SAH	42 (40.7%)
DM	8 (7.7%)
COPD	21 (20.3%)
Previous stroke	11 (10.5%)
Preoperative definitive pacemaker	1 (0.1%)
**NYHA Functional class**	
Class 1	16 (15.5%)
Class 2	63 (61.5%)
Class 3	17 (18%)
Class 4	7 (5%)
EuroSCORE (mean ± SD)	4.99±2.06
**Type of AF**	
Paroxysmal	13 (12%)
Persistent	8 (8%)
Permanent	82 (80%)
Previous AF time ( mean ± SD )	39.9±4.68 months
Left atrial size ( mean ± SD )	5.6±0.8 cm
LV ejection fraction ( mean ± SD )	58.34%±11.65 (Teichholtz )
**Medications**	
Oral anticoagulant	39.5%
LMWH	18.6%
Beta-blockers	27.8%
Digitalis	23.2%
Antiarrhythmics	6.9%
Diuretics	65%
ACE inhibtor	23%
ARBs	6.9%

NYHA=New York Heart Association; AF=atrial fibrillation; LV=left ventricle; cm=centimeter; LMWH = low molecular weight heparin; ACE = angiotensin-converting enzyme; ARBs = angiotensin receptor blockers

The results are presented as mean ± standard deviation for continuous variables and percentage for categorical variables. The evaluation of the effect of ablation surgery was performed using Student's t-test for the parametric data, Chi-square and Wilcoxon tests for the others. Univariate and multivariate regression analyzes were performed to evaluate predictive factors for AF recurrence. Survival curves and event-free survival were constructed using the Kaplan Meyer method. Values of probability less than 0.05 were considered statistically significant.

## RESULTS

**Fig. 3:**
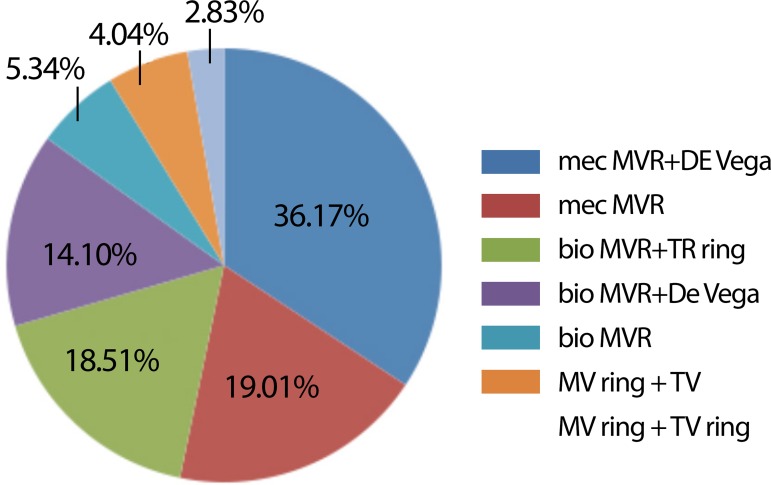
Types of surgeries performed. mec MVR=mechanical mitral valve replacement; De Vega= Tricuspid repair using De Vega technique; bio MVR=Biological mitral valve replacement; TR ring=Tricuspid repair with ring; MR ring=Mitral repair with ring; TR=Tricuspid repair

Surgeries performed are shown in Figure 3.

**Fig. 4:**
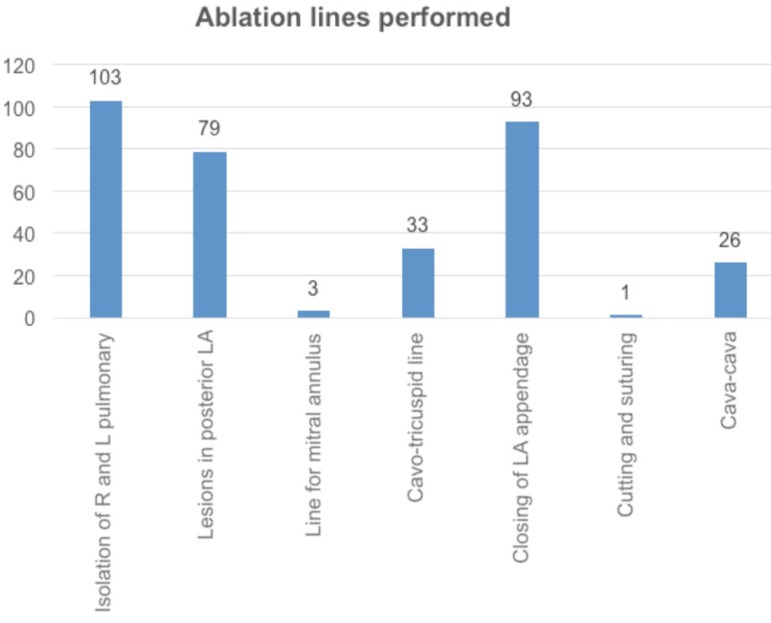
Ablation lines performed. Results expressed in absolute numbers. R=right; L=left; LA=left atrium

The operative variables are listed in Table 2 and the ablation lines performed in Figure 4:

**Table 2:** Perioperative data.

CPB time (min)	125.47±30.52
Myocardial ischemia time (min)	105.85±28.34
Type of ablation	
Unipolar	20.19%
Bipolar	79.81%

CPB=cardiopulmonary bypass; Min=minutes

Eight (7.8%) patients died in the first 30 postoperative days and were considered as procedure-related deaths. The causes of death in this period were: atrioventricular disjunction in two patients, hemorrhagic shock in one patient, septic shock in two patients, cardiogenic shock in two patients and cardiogenic shock associated with adult respiratory distress syndrome in one patient. There were no deaths during the one-year follow-up period of the patients who were discharged from the hospital.

The occurrence of a new stroke was 1%: one patient presented neurological event 17 days after surgery, who is still hospitalized. The heart rate, at the time of the event, was the same as of AF. The symptomatology reversed completely in 7 days. There were no other neurological events during the one-year follow-up period.

**Fig. 5:**
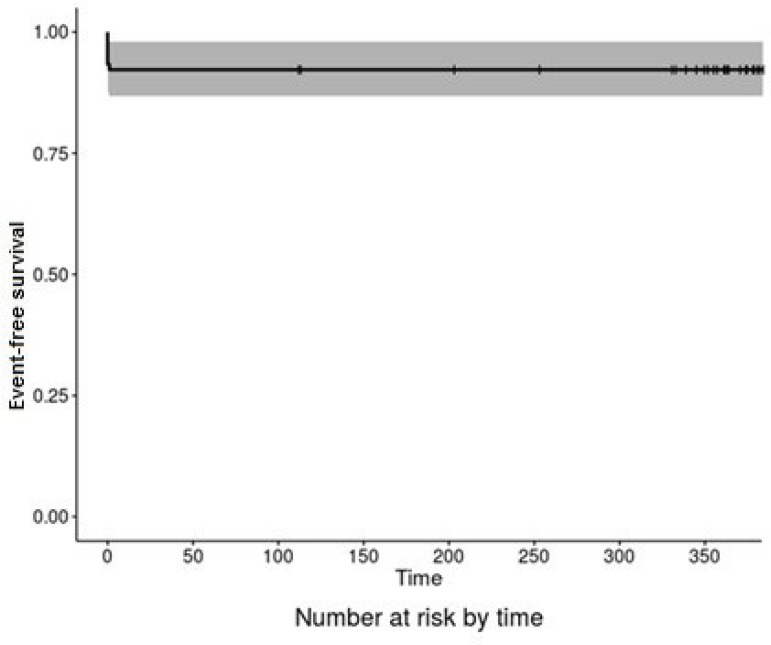
Actuarial curve for event-free survival (stroke and death).

Figure 5 shows the actuarial curve for event-free survival (death and stroke) one year after surgery.

**Fig. 6:**
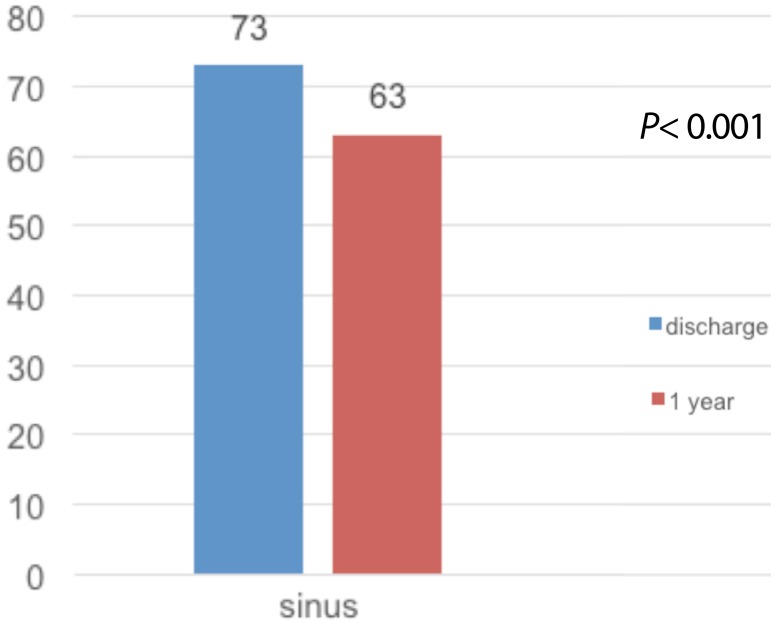
Cardiac rate at discharge and at the end of the 1-year postoperative follow-up period.

All survivors were followed-up for at least one year. At the time of hospital discharge information on heart rate was present in the medical records of the 95 (100%) surviving patients. Among them, 70 (73.7%) patients were in sinus rhythm, 22 (23.1%) patients in the rhythm of AF and 3 (3.1%) patients in pacemaker rhythm, one of whom already had a definitive pacemaker before surgery (Figure 6). Two (2%) patients required a definitive new pacemaker in the immediate postoperative period.

**Fig. 7:**
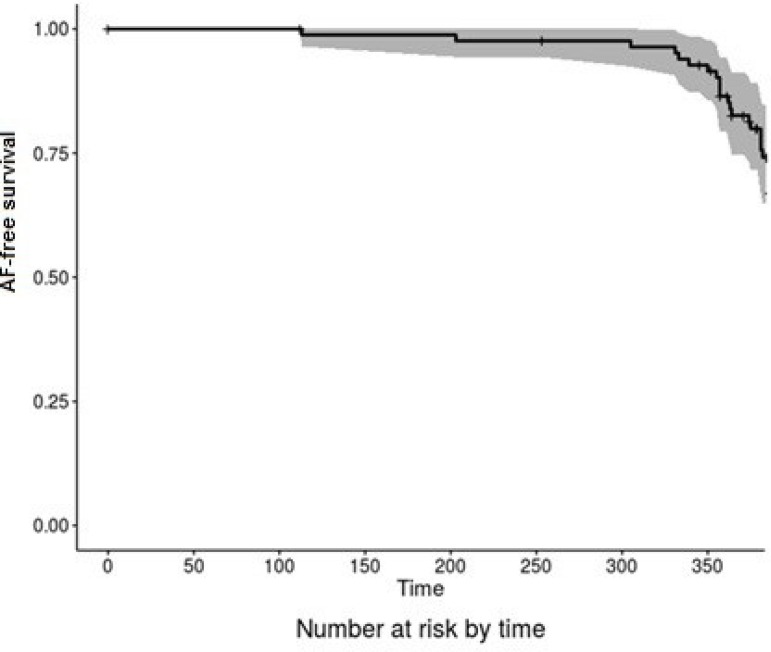
Actuarial curve for AF-free survival.

The one-year follow-up was performed in 100% of them (95 patients). A total of 388 electrocardiograms were analyzed in the postoperative follow-up period (3.3 electrocardiograms per patient) and 81 3-channels 24 hours Holter (0.8 Holter/patient). At the end of the one-year-follow-up period, 60 (63.1%) patients were in sinus rhythm, 18 (18.9%) patients were in AF, 10 (10.5%) were in right atrial flutter and pacemaker rhythm was present in 7 (7.3%). Figure 7 shows the actuarial AF-free survival curve of this population in the first year of the follow-up period. Figure 6 compares the prevalence of sinus and non-sinus rhythm between the time of hospital discharge and the end of the one-year-follow-up period.

In the multivariate analysis by binary logistic regression, three variables were associated with the occurrence of sinus rhythm during the one-year-follow-up period: sinus rhythm at discharge, left atrium size and use of a tissue ablation device by bipolar radiofrequency (Table 3).

**Table 3:** Multivariate analysis for sinus rhythm reversion one year after surgery.

Variables in the model	β	Standardized coefficient	95% Confidence Interval for the Settlement Ratio (SR)	
		Lower limit	SR	Upper limit
Constant	6.758			
Sinus rhythm on discharge**	-3.381	0.08	0.034	0.142
Size of the left atrium+	-0.840	0.209	0.432	0.803
Type of ablation^1^***	-2.079	0.032	0.125	0.483

1 Unipolar and Bipolar. R squared=0.494;

** *P*<0.001;

+ *P*=0.003;

*** *P* = 0.024.

Sinus rhythm at discharge increases by 29 times the chance of remaining in sinus rhythm in one year. The smaller the left atrium size the greater the chance of remaining in sinus rhythm in one year (2.3 times). The use of a tissue ablation device by bipolar radiofrequency increases this chance by eight times, when compared to the use of unipolar radiofrequency.

As left atrial size was the only continuous variable related to the chance of being or not in sinus rhythm in the 1-year follow-up period of these patients, the ROC curve was evaluated to try to define the most accurate left atrial size for determining the chance of being or not in sinus rhythm at the end of the one-year follow-up period of this population.

The cut-off point of left atrium size performed through efficiency was 5.35 cm.

## DISCUSSION

The pathophysiological mechanism of atrial fibrillation is not fully understood. The predominant 20^th^ century concept of multiple and chaotic atrial re-entry circuits following variant lines of conduction block was challenged by the observation that in some patients without structural heart disease the arrhythmia could only arise from a defined focus (generally emerging from the pulmonary veins)^[24]^. The two concepts seem to explain different forms of AF, the latter probably accounting for up to 80% of cases of paroxysmal AF in structurally normal hearts^[25]^. However, as the disease progresses, the atria grow and become more fibrous. AF outbreaks can then multiply and any treatment becomes less effective. Similarly, AF associated with mitral disease is associated with volume or pressure overload in the left atrium making it the main substrate of the arrhythmia. In cases of rheumatic disease^[10]^ the chronic inflammatory process itself may be responsible for the development of atrial fibrosis, even in the absence of valve dysfunction.

The curative ablation of AF has two mechanistic objectives: a) to remove all the triggers that could initiate or perpetuate the arrhythmia; b) alter the conduction properties of the atria so that AF cannot be sustained even if initiated. Only two approaches can modify the atrial substrate^[26]^. The first, through the creation of linear transmural lesions that connect two anatomical structures and form a conduction block (interrupting the re-entry circuits that perpetuate the arrhythmia). The second approach would involve reducing the amount of viable tissue^[24]^. The possible disadvantage of this approach would be the reduction of the potential of atrial contractility^[27]^.

The clinical objectives of performing an ablation of AF are to reduce or abolish symptoms (palpitation or symptoms of heart failure), to improve left ventricular function by restoring electrical and mechanical atrial systole, and ultimately to reduce the risk of stroke).

Cox Maze III surgery, developed in 1992, after long experiments on canine models^[16]^, has proven highly effective in addressing both the theoretical pathological mechanisms of arrhythmia described above and in achieving the clinical objectives of rate control (85% to 97% of long-term sinus rhythm)^[14]^ and prevention of stroke (incidence of 0.3% in 12 years^[4]^). Cutting and suturing biatrial lesions were performed by isolating the pulmonary veins, communicating with the mitral ring and venous sinus, in addition to several lesions in the right atrium. The major limitation of this method was its complexity and time for execution since all the lesions were performed by cutting and suturing method. Its diffusion in the surgical environment was always very limited. The advent of new forms of energy that could recreate transmural lesions safely, effectively, and rapidly led to a greater applicability of the method in several countries. Bipolar radiofrequency is released by a clamp that envelopes the endocardial tissue into the epicardium and has the ability to detect the moment the lesion becomes transmural^[28]^. The unipolar radiofrequency is released by a pen-like instrument and must be applied in the endocardium point-by-point. This instrument does not have the ability to detect the transmurality of the lesion.

The use of these methods of surgical ablation in Brazil is still very limited when compared to official data from the Thoracic Surgery Society (STS) of North America^[29]^. Between 2005 and 2010 more than 85,000 surgical ablations were performed associated with main cardiac surgery and another 5,000 isolated ablations. About 40% of AF patients who underwent cardiac surgery received some form of ablation. We do not yet have data from the Brazilian practice.

In this study, we chose to study a homogeneous group of patients with mitral disease of rheumatic origin. Most of them (82%) presented functional tricuspid insufficiency with need for associated tricuspid repair. About 80% of the individuals presented the permanent form of the arrhythmia and the mean size of the left atrium was 5.5 cm. All these characteristics lead us to the conclusion that this population had advanced cardiac structural disease, which increases surgical risk, impedes perioperative care and decreases the chance of successful atrial fibrillation ablation, as discussed above.

The mean surgical risk estimated by the EuroSCORE standard places this population in an intermediate risk zone for high risk. Therefore, in percentage numbers, the risk of death in 30 days for this population between 5 and 10% can be inferred. The occurrence of 8 (7.8%) deaths during the 30-day postoperative period seems to be within the confidence interval expected by this surgical risk calculation tool, inferring that there was no negative impact of completion of concomitant surgical ablation in the operative mortality in this population. In a meta-analysis of randomized controlled trials, used to evaluate the surgical treatment of fibrillation in cardiac surgery performed by Phan et al.^[30]^ no significant difference between surgical ablation *versus* non-ablation in terms of mortality was observed (OR, 1.05, 95% CI 0.66 to 1.68, *P*=0.83) and neurological events (OR, 0.86, 95% CI 0.37 to 2.04, *P*=0.74).

The 1-year mortality found in our study was 7.8%. It is noteworthy that all patients died within the 30-day postoperative period. In a recent multicenter, randomized study by Gillinov et al.^[31]^, where patients with permanent AF undergoing mitral valve surgery concomitant to ablation were analyzed, no significant difference in the one-year mortality was observed between the mitral valve ablation group (6.8%) and the control group of isolated mitral surgery (8.7%) (*P*=0.57).

In our study, the occurrence of stroke before surgery was 10.6% (11 patients) and after the 1-year follow-up period was 1.15% (1 patient). The study by Gillinov et al.^[31]^ shows the occurrence of 3% of stroke one year after the surgical procedure.

Our analysis also showed impact of left atrial size and occurrence of sinus rhythm in 1 year which correlates with the descriptions in the literature. The rate of sinus rhythm patients after surgical ablation of AF at hospital discharge of 73% and, 63% during the 1-year follow-up period, is compatible with the current series reported in rheumatic patients^[20]^.

In our series, an interesting non-significant difference in sinus rhythm was found one year after surgery when bipolar (65%) and unipolar (40%) energy were used (*P*=0.07). In the multivariate analysis, however, the use of tissue ablation devices by bipolar radiofrequency was associated with an 8-fold greater chance of being in sinus rhythm one after the procedure. The results associated with the use of uni- or bipolar radiofrequency for surgical ablation of AF are conflicting in the literature: Chen et al.^[29]^ in a recent series of 324 patients undergoing cardiac surgery for rheumatic valve disease (mitral, aortic and tricuspid) with concomitant AF ablation using bipolar tweezers found sinus rhythm in 87% of patients after the 1-year follow-up period. On the other hand, Pinho-Gomes et al.^[32]^ in a mixed series of patients undergoing mitral surgery of rheumatic and degenerative etiology, using only unipolar clamp, achieved sinus rhythm in two years in only 40% of the patients. Lazoupoulos et al.^[33]^ report a series of 93 patients undergoing ablation concomitant to mitral surgery in which both types of radiofrequency energy were used. After a 22-month follow-up period, 69% of the patients were in sinus rhythm, but the type of energy used was not a determinant of success or failure. The great variation of therapeutic success (40% to 89%) observed in the literature^[19,34]^ is due to different surgical populations, use of different forms of energy and heterogeneity in the choice of lesion lines performed. Although unipolar radiofrequency energy is considered useful in the confection of surgical ablation^[22]^, some disadvantages in relation to bipolar clamps are observed^[29]^, especially the longer time necessary to perform the lesions and the impossibility of detecting its transmurality.

In our Institution, the rheumatic etiology leads the indications of mitral valve surgery. It is important to bear in mind that this is a risk factor for therapeutic failure of AF ablation^[35,36]^. In this group of patients, attention is drawn to the appearance of therapeutic failure due to right atrial flutter in 10% of the patients. A better understanding of the pathophysiological mechanisms of atrial flutter in the postoperative period may help to minimize this therapeutic failure, possibly when considering the right atrial lines in all patients, as discussed by Cox in his original study on the results of classic cut and suture procedure.

### Limitations

Retrospective analysis with information collection in non-digitized medical records. There is no institutional protocol for performing the surgical ablation lines that were performed by several teams of different surgeons. Use of different devices related to the surgeon's preference and availability in the Institution. Unstructured follow-up care protocols for the management of postoperative atrial fibrillation. Use of electrocardiogram and Holter (less than 1 Holter/patient) only for the detection of AF.

## CONCLUSION

Atrial fibrillation ablation surgery combined with a surgical approach of the rheumatic mitral valve has been shown to be safe, with excellent survival and stroke-free survival during the one-year follow-up period. The majority of patients undergoing combined surgery were discharged from hospital at sinus rhythm and this finding was a predictor of maintenance of sinus rhythm during the one-year follow-up period. Increased left atrium and use of tissue ablation device by unipolar radiofrequency were associated with a lower chance of being in sinus rhythm one year after surgery.

**Table:** 

Authors' roles & responsibilities	
EKC	Substantial contributions to the conception or design of the work; or acquisition; final approval of the version to be published
ASC	Substantial contributions to the conception or design of the work; or acquisition; final approval of the version to be published
AJOM	Substantial contributions to the conception or design of the work; or acquisition; final approval of the version to be published
LSC	Substantial contributions to the conception or design of the work; or acquisition; final approval of the version to be published
ETM	Substantial contributions to the conception or design of the work; or acquisition; final approval of the version to be published
CW	Substantial contributions to the conception or design of the work; or acquisition; final approval of the version to be published
ONB	Substantial contributions to the conception or design of the work; or acquisition; final approval of the version to be published
AO	Substantial contributions to the conception or design of the work; or acquisition; final approval of the version to be published
